# Epigenetic signatures in antidepressant treatment response: a methylome-wide association study in the EMC trial

**DOI:** 10.1038/s41398-022-02032-7

**Published:** 2022-07-07

**Authors:** J. Engelmann, L. Zillich, J. Frank, S. Wagner, M. Cetin, D. P. Herzog, M. B. Müller, A. Tadic, J. C. Foo, L. Sirignano, D. F. Braus, N. Dahmen, S. Sordon, M. Riemenschneider, C. Spaniol, G. Gasparoni, M. Rietschel, S. H. Witt, K. Lieb, F. Streit

**Affiliations:** 1 Department of Psychiatry and Psychotherapy, University Medical Center, Mainz, Germany; 2 Translational Psychiatry, Department of Psychiatry and Psychotherapy & Focus Program Translational Neuroscience, University Medical Center, Mainz, Germany; 3 Department of Genetic Epidemiology in Psychiatry, Central Institute of Mental Health, Medical Faculty Mannheim, Heidelberg University, Mannheim, Germany; 4 Department of Psychiatry, Psychotherapy and Psychosomatics, Dr. Fontheim Mentale Gesundheit, Liebenburg, Germany; 5 Department of Psychiatry and Psychotherapy, Vitos Rheingau, Eltville, Germany; 6 Department of Psychiatry, Psychosomatics and Psychotherapy, Burghof-Klinik, Rinteln, Germany; 7 Department of Psychiatry and Psychotherapy, Saarland University Hospital (UKS), Homburg, Germany; 8 Department of Genetics, Saarland University, Campus A2 4, 66123 Saarbrücken, Germany; 9 Center for Innovative Psychiatric and Psychotherapeutic Research, Biobank, Central Institute of Mental Health, Medical Faculty Mannheim, Heidelberg University, Mannheim, Germany

**Keywords:** Pharmacogenomics, Predictive markers

## Abstract

Although the currently available antidepressants are well established in the treatment of the major depressive disorder (MDD), there is strong variability in the response of individual patients. Reliable predictors to guide treatment decisions before or in an early stage of treatment are needed. DNA-methylation has been proven a useful biomarker in different clinical conditions, but its importance for mechanisms of antidepressant response has not yet been determined. 80 MDD patients were selected out of >500 participants from the Early Medication Change (EMC) cohort with available genetic material based on their antidepressant response after four weeks and stratified into clear responders and age- and sex-matched non-responders (*N* = 40, each). Early improvement after two weeks was analyzed as a secondary outcome. DNA-methylation was determined using the Illumina EPIC BeadChip. Epigenome-wide association studies were performed and differentially methylated regions (DMRs) identified using the comb-p algorithm. Enrichment was tested for hallmark gene-sets and in genome-wide association studies of depression and antidepressant response. No epigenome-wide significant differentially methylated positions were found for treatment response or early improvement. Twenty DMRs were associated with response; the strongest in an enhancer region in *SORBS2*, which has been related to cardiovascular diseases and type II diabetes. Another DMR was located in *CYP2C18*, a gene previously linked to antidepressant response. Results pointed towards differential methylation in genes associated with cardiac function, neuroticism, and depression. Linking differential methylation to antidepressant treatment response is an emerging topic and represents a step towards personalized medicine, potentially facilitating the prediction of patients’ response before treatment.

## Introduction

Major depressive disorder (MDD) is one of the most common, burdensome, and costly mental disorders worldwide [1, 2]. Although currently available pharmacological treatments of MDD are well established and safe, there is a strong variability in antidepressant treatment response and considerable number of depressed patients do not respond to the first antidepressant administered, requiring optimization of antidepressant pharmacotherapy [3–5]. Due to the unpredictable treatment outcome, there is a vital need to identify reliable predictors of antidepressant response to guide treatment decisions. In clinical studies, early improvement, defined as a decrease in depressive symptomatology after two weeks, is considered the most consistent clinical predictor of antidepressant response [6]. However, research has not identified any clinical and biological predictor of sufficient clinical utility to inform the selection of a specific antidepressant agent for an individual depressed patient to date [7, 8].

MDD is moderately heritable, with heritability estimates from twin studies ranging between 30 and 40% [9]. In a recent genome-wide association study (GWAS), which investigate the association of common genetic variants with depression, 102 independent genome-wide significant variants contributing to disorder risk were identified, and the phenotypic variance explained by all investigated SNPs (i.e., SNP-heritability) was estimated to be 8.9% [10]. During the last few decades, scientific knowledge about the genetic background of depression has increased steadily and pharmacogenetic approaches have broadly been investigated to identify genetic variation contributing to individual treatment response in order to improve response prediction. A recent GWAS of antidepressant treatment response in 5151 depressed patients did not yield any genome-wide significant finding, but showed that genetic variation explained around 13% of variance in the total meta-analysis and 20–40% of variance within each cohort [11]. It has to be noted that the included samples showed high heterogeneity, and because of the relatively small sample size compared to other GWAS in psychiatric genetics, analyses were not stratified for diagnosis, drug, or drug dosage.

In addition to genetic variation, epigenetic alterations, specifically DNA-methylation, i.e., the addition of a methyl-group to a cytosine nucleobase at the 5′ position in CpG dinucleotides, can influence gene expression and may induce a wide range of potentially long-lasting changes at the cellular and systems function level [12, 13]. The investigation of epigenetic variation represents a promising approach to investigate the biological mechanisms underlying depression as well as the response to antidepressant treatments. Previous epigenetic investigation of antidepressant treatment response focused mainly on well-described candidate genes like *BDNF, NR3C1*, and *FKBP5* (reviews: [14–16]), but results are inconclusive. Epigenome-wide association studies (EWAS) represent a promising approach to identify new biological mechanisms underlying individual differences in the context of antidepressant response. Specific methylation patterns might both be indicative of general treatment resistance, as well as of the propensity to respond to a specific medication. By that, identified methylation signatures might guide clinical decisions in the future. Recent EWAS have investigated the association of DNA-methylation with depression [17–20], as well as with antidepressant use [21]. Only one of the mentioned studies [20] yielded epigenome-wide significant results, although the two identified CpG-sites were not annotated to nearby genes, which makes functional interpretation difficult. Results from EWAS can, similar to those from GWAS, be summarized in methylation risk scores (MRS) and a recent study has shown that a MRS for depression was able to explain ~2% of variance in depression and provided additional prediction to polygenic risk scores [22].

So far, only two studies have investigated genome-wide differences in baseline DNA methylation between responders and non-responders to pharmacological antidepressant treatment. Ju and colleagues revealed several CpG-sites differentially methylated between responders (*N* = 82) and non-responders (*N* = 95) to eight weeks of escitalopram treatment, which were also associated with gene expression differences between both groups [23]. The study highlighted a differential methylated position (DMP) located in the *CHN2* gene, which was most significantly associated with mRNA expression and was replicable in an external cohort with a similar treatment [23]. The second study by Martinez-Pinteno et al. [24] identified 21 differentially methylated DMPs between responders and non-responders (*N* = 11, each) to eight weeks of fluoxetine treatment in a cohort of depressed children and adolescents. The *Ras Homolog Family Member J* (*RHOJ*) gene, encoding signaling molecules in the regulation of cytoskeletal organization, showed four significantly hypermethylated CpG-sites in non-responders [24]. These findings highlight studying baseline methylation differences as a predictor of antidepressant response as a promising approach to investigate interindividual differences in antidepressant response. Further investigations of DNA methylation signatures between later responders and non-responders are needed to extend and solidify the gained knowledge. Perspectively, studies identifying epigenetic markers associated with therapy response, might help to understand the underlying mechanisms and indicate new targets to modulate therapy response, or serve as a marker to predict response in a precision medicine way.

The primary aim of this study was to identify epigenetic signatures associated with antidepressant treatment response, by testing baseline differential methylation between responders and non-responders after four weeks of antidepressant treatment. Patients were enrolled from a large well-characterized antidepressant trial, allowing us to carefully select clear responders and sex- and age-matched non-responders. As a secondary aim, we explored the DNA-methylation signatures of early improvement in the same sample.

## Patients and methods

### Sample

This investigation is a secondary analysis of 80 MDD patients, who have participated in the “Randomized clinical trial comparing an early medication change (EMC) strategy with treatment as usual (TAU) in patients with MDD—the EMC trial” (ClinicalTrials.gov NCT00974155). A total of 889 depressed patients were enrolled between 2009 and 2014 in this trial. Genetic material at baseline was available for 560 patients, of which the 40 most clear responders and sex- and aged-matched non-responders were selected. The selection was based on their treatment response, measured with the Hamilton Rating Scale for Depression – 17 items (HAMD_17_) to antidepressant study medication after four weeks. In addition, the course of depression severity was considered in weekly intervals from baseline to week 4. The non-responders showed no improvement in depressive symptomatology despite four weeks of antidepressant treatment; in the group of responders, depressive symptomatology decreased steadily and patients showed a complete remission after four weeks (see also *Study Procedures*). Details of the EMC study protocol have been described previously [25–27] and the treatment algorithm can be openly accessed by https://trialsjournal.biomedcentral.com/articles/10.1186/1745-6215-11-21. In summary, the EMC trial was a multi-center, randomized, controlled clinical trial investigating whether patients with non-improvement after 14 days of escitalopram treatment take advantage to an early medication change (EMC: change to venlafaxine from day 14 onwards followed by an augmentation with lithium after again non-response at day 28) compared to patients treated according to current guideline recommendations (TAU: continuing escitalopram for two more weeks and switching to venlafaxine at day 28). All participants gave their written informed consent to participate in the study after a complete and extensive description. Study procedures were approved by the local ethics committee of the Landesärztekammer Rheinland-Pfalz and are compliant with the Code of Ethics of the World Medical Association (Declaration of Helsinki) in its current version.

### Study procedures

Diagnoses were based on the German Version of the Mini International Neuropsychiatric Interview (M.I.N.I. [28]) and the Structured Clinical Interview for DSM-IV Axis II Personality Disorders (SCID-II [29]). The socio-demographic and clinical characteristics, such as previously diagnosed cardiovascular or metabolic diseases and smoking, were assessed relying on patients’ self-reports. Depression severity was measured weekly from baseline to day 56 by the HAMD [30] by trained and blind raters [31]. Morning blood samples were obtained weekly before the first medication intake in fasting patients. Antidepressant premedication was—if necessary—washed out after inclusion and before baseline visit. Therefore, no antidepressant medication was used by the participants, when blood was drawn for the present DNA methylation analysis. The antidepressant treatment according to study protocol was 20 mg escitalopram from baseline to day 14, followed by a predefined treatment algorithm. Other medications were administered to treat depression-associated symptoms (e.g., insomnia) or adverse drug reactions (e.g., agitation or anxiety) with short-acting hypnotics (zolpidem or zopiclone), low potency antipsychotic drug pipamperone, histamine-receptor antagonist promethazine in standard doses as well as benzodiazepines in a dose-equivalent up to 15 mg diazepam per day was allowed. The main outcome parameters were: a) response, defined as decrease of 50% during four weeks of treatment, and b) early improvement, defined as a decrease of depression severity of at least 20% from baseline to day 14 [6].

### DNA-methylation

DNA was extracted from whole blood, collected at baseline and before the first intake of the antidepressant study medication, using the QIAamp DNA Blood Midi Kit from Qiagen (Qiagen, Hilden, Germany). The genomic DNA samples were stored at −20 °C. Responders and non-responders were matched based on age and gender and the DNA from the matched samples were randomized and pipetted on processing plates. 500 ng genomic DNA were bisulfite converted using the EZ-96 DNA methylation gold kit (Zymo research, Irvine, USA). Epigenome-wide methylation levels were determined using the Illumina HumanMethylationEPIC Beadchip and Illumina HiScan array scanning systems (Illumina, San Diego, CA).

### Data preprocessing and quality control

The R statistical environment, version 3.6.1, was used for all data preprocessing and analysis steps. We used an updated version of the CPACOR-pipeline to extract methylation data from raw intensity data and performed quality control [32]. Thresholds for sample removal were: (i) DNA quality was not sufficient (missing rate > 0.10) or (ii) a discrepancy between methylation-based and phenotypic sex emerged. Thresholds for probe removal were: (i) the call-rate was insufficient (<0.95), (ii) SNPs with a minor allele frequency >0.10 were located in the probe sequence, (iii) the probes were located on the X or Y chromosome. After quality control all 80 samples remained. After filtering, 706,677 out of 843,232 sites were available for analysis.

### Statistical analysis

Differences in clinical and sociodemographic characteristics between responders and non-responders were calculated by t-tests for independent variables or Chi^2^-tests, depending on the level of measurement.

Methylation values were log-transformed (base2) and included as dependent variables in the association analyses [33]. Principal component analysis was performed to extract signals of the internal control probes of the EPIC array and the resulting first ten principal components were included in all analyses to control for batch effects and technical quality. Additionally, the chip number and position on the chip were included. Cell-type heterogeneity was accounted for by estimating the cell counts based on the methylation data [34]. This approach results in six estimates, which roughly sum up to one. To avoid multicollinearity in the EWAS, variance inflation factors were calculated for each cell count estimate. The estimated granulocyte count was subsequently removed from further analyses. For two participants, data on smoking was not available, and their smoking status was therefore estimated based on a validated set of nine CpG-sites [35]. Probability of smoking was calculated using the predict function in R with the nine CpG sites from Maas and colleagues [35], as well as sex and age as covariates. Participants with a probability above 50% were classified as smokers and those with a probability below 50% as non-smokers.

#### Epigenome-wide association analysis

Tests of single site methylation differences between responders and non-responders were performed with linear models, adjusting for sex, age, smoking, standardized cell counts, and the first ten principal components of the internal control probes. Additionally, all analyses were run with early improvement after two weeks as a secondary outcome. Correction for multiple testing was applied using the Benjamini–Hochberg (FDR) correction and the resulting values are reported as *q-*values. CpG-sites were annotated using the manufacturer’s manifest (http://webdata.illumina.com.s3-website-us-east-1.amazonaws.com/downloads/productfiles/methylationEPIC/infinium-methylationepic-v-1-0-b4-manifest-file-csv.zip; downloaded on 10th of August 2018).

#### Differentially methylated regions (DMRs)

The comb-p algorithm was applied to identify DMRs. Comb-p accounts for autocorrelation between tests of adjacent methylation sites and combines these sites, in a given window, to regions of enrichment [36]. In the present study the settings were: Seed-*p* value < 0.01, minimum of 2 probes, sliding window 500 bp. Correction for multiple testing was applied using the Šidák correction as implemented in comb-p.

#### Gene-set enrichment analysis

missMethyl [37] was used for functional analysis to test differentially methylated CpG-sites overrepresented in Hallmark gene-sets. Sites with a threshold of *p*_nominal_ < 0.001 were included and the Hallmark gene-set collection (MSigDB Version 7.1), which consists of 50 gene-sets representing specific well-designed biological states or processes, was used as reference [38]. missMethyl controls for several potential confounders, such as probe number bias, which is the increased likelihood of a gene being differentially methylated, if more probes cover the gene, and multi-gene bias, since probes can be annotated to more than one gene.

#### GWAS-enrichment-analysis

Gene-sets consisted of the genes to which CpG-sites with an uncorrected *p*-value < 0.001 in the EWAS were annotated to. Two gene-sets were created, one for early improvement and one for treatment response. Gene-set enrichment was tested in results of the two recent genome-wide association studies described in the introduction: one of antidepressant treatment response (*N*_remission_ = 1852, *N*_non-remission_ = 3299) [11] and one of MDD including PGC and UKB samples (*N*_cases_ = 246,363, *N*_controls_ = 561,190) [10]. This test was performed using Multi-marker Analysis of GenoMic Annotation (MAGMA) [39].

#### GWAS Atlas/PheWAS

We performed a Phenome-wide association study (PheWAS) for each gene that was implicated by the DMR analysis, using the GWAS Atlas tool (https://atlas.ctglab.nl/PheWAS).

#### Overlap with MDD EWAS

Regression coefficients of the top 100 differentially methylated positions associated with response and early improvement in the present study were extracted from the summary statistics (MWAS2) of a large MDD EWAS in Generation Scotland [20]. Here, we used the summary statistics from the MWAS2, in which a complex model using the OmicS-data-based Complex trait Analysis tool was tested, in which *M* values were adjusted for several confounding variables and cell counts were fitted as fixed effects [20].

## Results

**Fig. 1:**
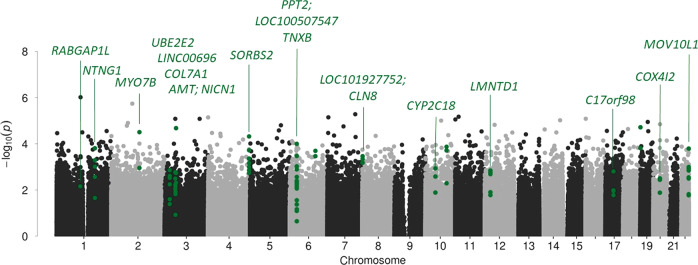
Mean Hamilton Rating Scale for Depression – 17 items (HAMD_17_) Score by response to treatment, light gray represents responder, dark gray non-responder. Error bars represent standard deviations.

The course of depression severity in responders and non-responders is shown in Fig. 1. Mean age (±SD) was 41.5 (±11.1) years, 58% of patients were women and depression severity at baseline was 22.3 (±4.1) points (HAMD_17_). As individuals were matched, there were no differences in age or sex distribution (all *p* ≥ 0.94). All patients were treated with 20 mg escitalopram from baseline to day 14. In responders, escitalopram was continued unchanged until day 28. In non-responders, 42.5% (17 of 40 patients) were switched to high-dose venlafaxine (225–375 mg) from day 14 onwards. For details of the clinical and sociodemographic characteristics see Table 1.

**Table 1:** Clinical and sociodemographic data.

	Total (*N* = 80)	Responders (*N* = 40)	Non-responders (*N* = 40)	*p*-value (group comparison)
Age – yrs (SD)	41.55 (11.09)	41.45 (10.83)	41.65 (11.49)	0.94^a^
Female	46 (57.5%)	23 (57.5%)	23 (57.5%)	1.0^b^
Male – (%)	34 (42.5%)	17 (42.5%)	17 (42.5%)	
Age at onset – yrs (SD)	33.48 (12.02)	36.00 (12.13)	30.95 (11.50)	0.06^a^
Duration of current episode – wks (SD)	30.38 (41.94)	28.15 (40.87)	32.60 (43.39)	0.64^a^
1st episode	28 (35%)	19 (48%)	9 (23%)	0.034^b^
Recurrent – (%)	52 (65%)	21 (52%)	31 (77%)	
Hamilton scores				
Baseline (SD)	22.33 (4.15)	22.83 (3.88)	21.82 (4.4)	0.28^a^
Day 14 (SD)	12.69 (8.83)	4.57 (2.26)	20.8 (4.21)	<0.001^a^
Day 28 (SD)	12.61 (10.50)	2.63 (1.76)	22.60 (3.97)	<0.001^a^
Smokers – (%)				0.35^b^
Yes	28 (36%)	16 (42%)	12 (30%)	
No	50 (64%)	22 (58%)	28 (70%)	
Cardiovascular disease – (%)				0.0504^b^
Yes	16 (20%)	4 (10%)	12 (30%)	
No	64 (80%)	36 (90%)	28 (70%)	
Metabolic disease – (%)				0.735^b^
Yes	10 (12.5%)	4 (10%)	6 (15%)	
No	70 (87.5%)	36 (90%)	34 (85%)	
Early improvement – (%)				
Yes	36 (45%)	39 (97.5%)	5 (12.5%)	
No	44 (55%)	1 (2.5%)	35 (87.5%)	

Continuous measures are presented as mean (standard deviation) and categorical measures as frequency (percent).

Notes: ^a^t test; ^b^χ² test; *SD* standard deviation, *wks* weeks; *yrs* years.

### Epigenome-wide association study

No epigenome-wide significant differentially methylated positions emerged for either treatment response or early improvement after controlling for multiple testing. The strongest association with response was observed with hypermethylation of cg02107110 in *WDR47* (*β* = 0.17, *p* = 9.59*10^−7^, *q* = 0.57). For early improvement, the strongest association was observed for cg04568295, which was annotated to *SIRT7* and *MAFG* (*β* = 0.11, *p* = 1.58*10^−6^, *q* = 0.86). Regression coefficients for the 100 DMPs showing the strongest association can be found in Supplementary Table S1 for response and S2 for early improvement.

### Differentially methylated regions (DMRs)

**Fig. 2:**
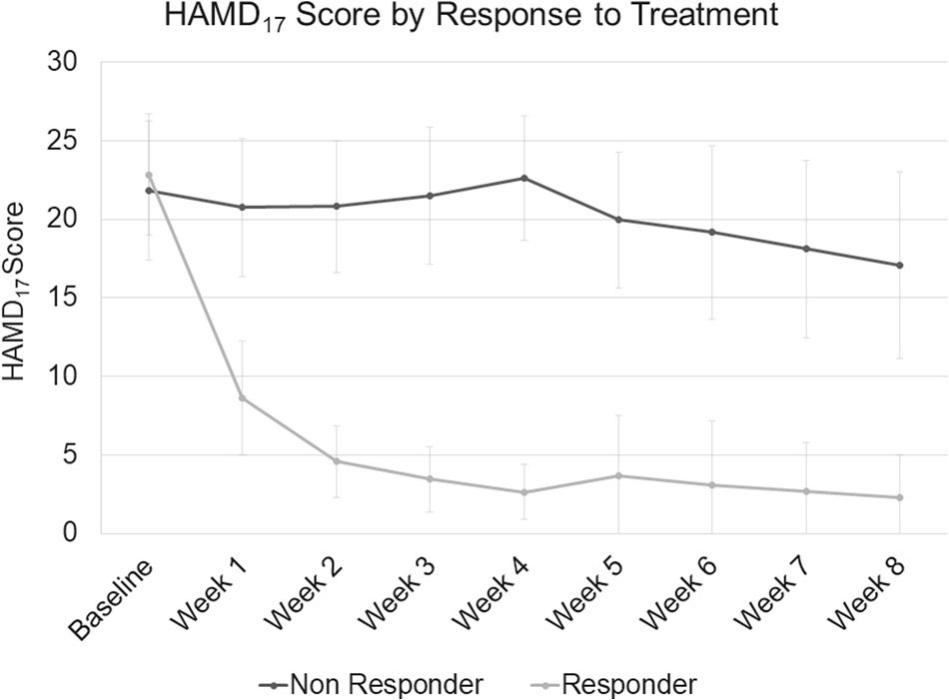
Differentially methylated CpG-sites and regions (green) associated with treatment response after four weeks.

The DMR analysis identified twenty DMRs associated with treatment response and eleven with early improvement, Table 2 lists the DMRs and Fig. 2 depicts the Manhattan plot of this analysis; DMRs are highlighted; results for early improvement are listed in Supplementary Table S3. The DMR showing the strongest association for both response to treatment and early improvement was annotated to *Sorbin And SH3 Domain Containing 2 (SORBS2)*, a protein coding gene. The DMR consists of eight CpG-sites, hypermethylated in the responder group, seven of which are part of an enhancer region of *SORBS2*, pointing towards a potential functional relevance.

**Table 2:** Differentially methylated regions associated with response after four weeks of treatment.

Chr	Start	Ende	*N* probes	*P*	Sidak *P*	Gene	Direction
4	186732837	186733061	8	5.84E-16	1.75E-12	*SORBS2*	+
6	32016214	32016427	8	2.49E-12	8.26E-09	*TNXB*	+
1	174844397	174844561	5	1.19E-09	5.11E-06	*RABGAP1L*	+
8	1713005	1713013	3	2.74E-08	2.42E-03	*LOC101927752;CLN8*	+
10	96442621	96442675	3	4.59E-08	6.01E-04	*CYP2C18*	+
3	23244051	23244131	6	4.84E-08	4.28E-04	*UBE2E2-AS1;UBE2E2*	+
19	996220	996374	2	5.30E-08	2.43E-04		*+*
12	25801455	25801622	5	5.41E-08	2.29E-04	*LMNTD1*	+
22	50528213	50528299	4	6.20E-08	5.10E-04	*MOV10L1*	+
3	52099522	52099562	3	7.73E-08	1.37E-03	*LINC00696*	*−*
22	50585229	50585401	4	1.58E-07	6.49E-04	*MOV10L1*	+
1	108023366	108023487	5	2.65E-07	1.55E-03	*NTNG1*	+
3	48632568	48632724	4	5.57E-07	2.52E-03	*COL7A1*	+
2	128366514	128366595	2	5.90E-07	5.13E-03	*MYO7B*	+
3	49459909	49460112	6	8.09E-07	2.81E-03	*AMT;NICN1*	+
20	30225681	30225852	4	8.45E-07	3.49E-03	*COX4I2*	+
6	116886276	116886350	2	9.22E-07	8.76E-03		*+*
6	32121355	32121523	8	1.44E-06	6.05E-03	*PPT2;LOC100507547*	+
17	36997563	36997732	4	3.75E-06	1.56E-02	*C17orf98*	*+*
10	45719880	45720041	2	5.02E-06	2.18E-02		*−*

Notes: *Chr* chromosome, − hypomethylation of DMR in responders, + hypermethylation of DMR in responders.

### Gene-set enrichment analysis

Results from the EWAS were most strongly overrepresented in the Hallmark gene-sets “apical surface” (*p* = 0.001, *q* = 0.078) and “myogenesis“ (*p* = 0.006, *q* = 0.142), although none of the terms remained significant after multiple testing correction. Results of the gene-set enrichment analysis can be found in Supplementary Table S4 for response and S5 for early improvement.

### GWAS-enrichment analysis

No significant enrichment of genes implicated by GWAS of MDD and antidepressant treatment response was observed (all *p* ≥ 0.12). Detailed results are listed in Table 3.

**Table 3:** Results of GWAS-enrichment analyses.

Outcome	GWAS	*N* Genes	Beta	SE	*P*
Response	ADR	560	0.01	0.036	0.388
	Depression	557	0.027	0.041	0.259
Early improvement	ADR	451	0.028	0.041	0.125
	Depression	448	0.06	0.049	0.12

Notes: *ADR* antidepressant treatment response, *SE* standard error.

### GWAS atlas/PheWAS

We performed a Phenome-wide association study of the genes in which the DMRs were identified. All traits which have been genome-wide significantly associated with the respective genes are listed in Supplementary Table S6a–m.

### Overlap with MDD EWAS

Regression coefficients of the top 100 differentially methylated positions associated with response and early improvement in the present study were extracted from the summary statistics of a large MDD EWAS in Generation Scotland [20]. These coefficients are listed for each of the top 100 CpG sites in Supplementary Tables S1 and S2. For response to treatment there was no systematic association of the CpG sites as effect sizes from both studies showed a null correlation (*r* = 0.01, *p* = 0.91) and a cross table of the direction of effects did not reveal systematic overlap. For early improvement, a small, but non-significant, positive correlation between effect sizes was observed (*r* = 0.18, *p* = 0.09) and 63% of effect estimates showed the same direction.

### Exploratory analysis

Genetic Variation in *SORBS2* has repeatedly been associated with cardiovascular and metabolic diseases. Therefore, we performed follow-up analyses to investigate whether there was an association between previously diagnosed cardiovascular diseases, such as high blood pressure or arrhythmia, metabolic diseases, such as diabetes or obesity, and treatment response. Descriptively, patients who responded to antidepressant therapy were less likely to have a previous diagnosis of a cardiovascular disease, but a chi-square test was not significant (Χ^2^(1) = 3.83, *p* = 0.0504). No association between metabolic diseases and treatment response was observed.

When we included previously diagnosed cardiovascular diseases in the EWAS regression model as a covariate, associations between treatment response and methylation of the DMR in *SORBS2* remained significant. Also, in a separate analysis, previously diagnosed cardiovascular diseases did not predict methylation in *SORBS2* (all *p* > 0.46).

## Discussion

The aim of the present study was to identify differential methylation signatures before treatment initiation associated with antidepressant treatment outcome in 80 MDD patients, who were part of a large randomized controlled trial. We focused our analyses on antidepressant response after four weeks and additionally investigated early improvement after two weeks of treatment. For both outcomes, several DMRs at baseline were observed, which may point to possible underlying mechanisms of differential response to antidepressant pharmacotherapy.

While the epigenome-wide association study did not yield findings remaining significant after correction for multiple testing on the single site level, the region-based analyses highlighted several CpG-sites as potentially relevant in antidepressant treatment response. The most strongly associated CpG-site for response was observed with hypermethylation of cg02107110 in the *WDR47* gene. WDR47 is a microtubule-associated protein and plays a role in neuronal regulation, brain development, and brain connectivity [40]. Regarding early improvement, the strongest associated CpG-site was found in cg04568295. DNA-methylation at this site has been associated with HbA1c-levels in type 1 diabetes [41].

The first epigenome-wide association study of antidepressant response by Ju and colleagues identified three DMPs before treatment between later responders (*N* = 82) and non-responders (*N* = 95). One DMP located in the *CHN2* gene could be replicated in a second cohort receiving the same antidepressant treatment [23]. However, the CpG-sites highlighted in *CHN2* were not available for analysis in the present study after quality control had been performed, and the DMP in the second highlighted gene (*JAK2)* which as available in our data set (cg08584037) was not significantly associated with response or early improvement in the present study (*p* > 0.05). It also has to be taken into account that the reported results of Ju and colleagues were not corrected for major drivers of differential methylation such as smoking [42]. The second study by Martinez-Pinteno and colleagues investigated baseline differences in DNA methylation between responders and non-responders (*N* = 11, each) and reported 21 significantly differential methylated CpG-sites associated with response to fluoxetine in adolescents. Within the two genes *RHOJ* and *OR2L13* (*Olfactory Receptor family 2 subfamily L member 13*), four and three DMPs were found between responders and non-responders [24]. These results were not replicated for antidepressant response in adults in the present study. It has to be noted that besides technical parameters such as the considered covariates, the studies differ in clinical aspects, which might influence the replicability of the results. One major aspect is the pharmacological treatment, with administration of a different medication in the study by Martinez-Pinteno et al. [24], and no EMC in the study by Ju et al. [23]. It is unclear to what degree the present results are specific to the applied medication regime. The epigenetic make-up influencing therapy response can be expected to differ for different antidepressants, and future studies and meta-analyses should take this into account.

The significant DMRs were found in genes associated with a variety of domains, such as psychiatric, skeletal, immunological, and metabolic traits. For example, genetic variation in *RABGAP1L* has been associated with the psychiatric traits ease of getting up in the morning [43], depressive affect [44], and neuroticism [45], but also metabolic traits such as BMI [46]. The strongest association was observed for a region in *SORBS2 (ARGBP2)*. This DMR was differentially methylated between responders and non-responders, as well as between patients who showed an early improvement after two weeks of treatment, and those who did not. *SORBS2* is a protein-coding gene, which encodes the sorbin and SH3 domain containing 2 protein. Interestingly, the identified DMR is in an enhancer region, which provides evidence for a potential functional mechanism. Genetic variation in *SORBS2* has previously been implicated in cardiovascular diseases [47], type II diabetes [48], and educational attainment [49] in European ancestry populations. A pharmacogenetic GWAS suggested an association of *SORBS2* in response to lithium treatment [50] and in the recently published EWAS by Zhu and colleagues, *SORBS2* was found as significant DMR associated with lifetime history of MDD in monozygotic discordant twins [18]. In addition, a review by Gharipour et al. highlighted *SORBS2* as one of three overlapping genes between mood disorders and obesity and formulated the hypothesis that hypermethylation in *SORBS2* might play a role in the co-occurence of both syndromes due to inflammation processes [51]. Based on the findings on *SORBS2* in cardiovascular and metabolic diseases, we performed additional exploratory analysis, including previously diagnosed cardiac and metabolic comorbidities of our MDD patients. Even after taking the comorbidities into account, the association between response and differentially methylation of *SORBS2* remained significant. Mechanistically, the *SORBS2* splice variant *neural Abelson-related gene-binding protein 2* (*nArgBP2*) could be of particular interest, because it is specifically expressed in neurons. The nArgBP2 protein is enriched at dendritic spines where it acts as a cytoskeletal adaptor protein [52]. Due to the specificity of nArgBP2 to excitatory synaptic inputs, dysregulation results in an excitatory/inhibitory imbalance that could contribute to the disease course in mood disorders [53] and also to the response to antidepressants.

Another DMR in *CYP2C18* identified in the present study is of particular interest as *CYP2C18* belongs to the cytochrome P450 super family, which is involved in metabolism of many drugs, and genetic variation in this gene has previously been associated with escitalopram treatment response [54]. The pharmacogenetic study by Braten and colleagues investigated novel CYP2C-haplotypes to improve genetic prediction of escitalopram metabolism. The presence of the two SNPs (i.e., rs2860840 (C > T: CYP2C18, 3’UTR) and rs11188059 (G > A: CYP2C18, intron5) was associated with a significantly lower serum concentration of escitalopram [54].

None of the gene-sets were significantly enriched for genetic variation identified in recent GWAS. This could be because the cut-off of *p* < 0.001 resulted in a relatively large gene-set, but a restriction to genes implied by DMR analysis did not yield significant findings either. Also, the GWAS of antidepressant treatment response, which is the most relevant for the present analysis, is relatively small in comparison to other GWAS on psychiatric phenotypes [11], and also the larger MDD GWAS is far from reliably identifying all associated variants [55]. In combination with the limited sample size of the present EWAS, the performed analyses might have been too limited in statistical power to detect an enrichment. Furthermore, the applied method is limited by mapping SNPs via chromosomal position to specific genes. It has been shown that besides those cis-regulatory effects, a substantial proportion of SNPs also act via trans-regulatory effects on more distal loci [56]. Another promising approach that could be pursued in future larger samples with available genetic and genotype data is to test the association of DNA methylation with polygenic risk scores calculated based on the respective GWAS [57].

A look-up of the top 100 CpG sites for treatment response in a large EWAS of MDD [20] did not reveal overlap between CpG sites associated with antidepressant treatment response and MDD. While this could point towards a specific DNA methylation signature of antidepressant treatment response, the results could again be in part attributable to low statistical power.

Overall, there is a wide correlation between the two investigated outcome parameters (response and early improvement) and the majority of differentially methylated CpG-sites were implicated in both outcomes. This can be simply explained from the patient sample investigated here in which 97.5% (*N* = 39) of responders at week 4 also showed an early improvement to antidepressant treatment at week 2. However, this is also in line with convergent evidence in literature from our own studies as well as a number of additional investigations that early improvement, defined as a 20% decrease of depressive symptomatology within the first two weeks, is one of the most consistent clinical predictors of later response to antidepressants [6, 58, 59], and that biological predictors of antidepressant treatment response may be identified already in the early course of treatment.

The major strength of our study is the selection of the investigated patients from the large well-characterized EMC trial, which enabled us to choose clear responders and non-responders and to match the two groups according to several criteria, such as age and sex. As antidepressants were washed out before the baseline blood sampling, confounding based on antidepressant use is minimal. In addition, we were able to control for potentially influencing factors such as smoking and to include concomitant cardiovascular and metabolic diseases in our analysis. However, we cannot exclude that further confounding lifestyle factors might have influenced our results, e.g., methylation in *SORBS2* has been linked to obesity [60].

Several limitations apply to the present study. The sample size is relatively small compared to case-control EWAS of MDD and therefore lacks statistical power. Even though we were able to identify DMRs associated with antidepressant treatment response, our results need to be confirmed in larger well-characterized MDD samples. Secondly, gene expression patterns could not be investigated in our sample, as the respective biomaterial is not available, limiting the possibility to draw conclusions about functional mechanisms. Thirdly, DNA methylation was assessed at baseline. While from a prediction perspective, it is important to identify pretreatment biomarkers of later therapy response, a longitudinal assessment could provide important insights into methylation changes associated with antidepressant treatment outcomes. Furthermore, methylation was assessed in peripheral blood samples and may potentially not reflect methylation in the brain of depressed patients.

In conclusion, we identified differential methylated regions associated with pharmacological antidepressant response in a well-characterized MDD study sample before treatment initiation. The DMR showing the strongest association was annotated to *SORBS2*, which has previously been described as an overlapping gene between mood disorders and obesity. *SORBS2* may therefore be a potential target gene enabling better understanding of mood disorders and additionally antidepressant treatment response, but confirmation in larger samples is needed. In summary, our results provide further evidence for the role of DNA methylation in patients’ response to antidepressant treatment. Exploring DNA methylation in larger and clinically well-characterized samples may lead enable the stratification into different response subtypes.

## Supplementary information

Supplementary Material

## Data Availability

Raw data and summary statistics for all analyses are available from the corresponding author on reasonable request.

## Code availability

Analysis code is available from the corresponding author on reasonable request.
